# State-of-the-art review of the clinical research on menopause and hormone replacement therapy association with Parkinson’s disease: What meta-analysis studies cannot tell us

**DOI:** 10.3389/fnagi.2022.971007

**Published:** 2022-10-20

**Authors:** Santiago R. Unda, Sabina Marciano, Teresa A. Milner, Roberta Marongiu

**Affiliations:** ^1^Department of Neurological Surgery, Weill Cornell Medicine, New York, NY, United States; ^2^Feil Family Brain and Mind Research Institute, Weill Cornell Medicine, New York, NY, United States; ^3^Harold and Milliken Hatch Laboratory of Neuroendocrinology, The Rockefeller University, New York, NY, United States; ^4^Department of Genetic Medicine, Weill Cornell Medicine, New York, NY, United States; ^5^Aligning Science Across Parkinson’s (ASAP) Collaborative Research Network, Chevy Chase, MD, United States

**Keywords:** Parkinson’s disease, menopause, hormone replacement therapy, risk, onset

## Abstract

The menopause is a midlife endocrinological process that greatly affects women’s central nervous system functions. Over the last 2 decades numerous clinical studies have addressed the influence of ovarian hormone decline on neurological disorders like Parkinson’s disease and Alzheimer’s disease. However, the findings in support of a role for age at menopause, type of menopause and hormone replacement therapy on Parkinson’s disease onset and its core features show inconsistencies due to the heterogeneity in the study design. Here, we provide a unified overview of the clinical literature on the influence of menopause and ovarian hormones on Parkinson’s disease. We highlight the possible sources of conflicting evidence and gather considerations for future observational clinical studies that aim to explore the neurological impact of menopause-related features in Parkinson’s disease.

## Introduction

Parkinson’s disease (PD) is the second most common neurodegenerative disorder, affecting over 6 million people worldwide, and this number is projected to double over the next 20–30 years (Poewe et al., 2017; GBD 2016 Neurology Collaborators, 2018). The disease is characterized primarily by the loss of dopaminergic neurons and presence of Lewy bodies (α-synuclein accumulation within surviving neurons) in the substantia nigra pars compacta (Poewe et al., 2017). The cardinal disease features consist in 4 main motor symptoms: bradykinesia, postural instability, rigidity, and tremor at rest (Poewe et al., 2017; Armstrong and Okun, 2020).

Evidence of a role for sex dimorphism in PD has increased over the last decades, showing that women, compared to men, present a lower PD incidence and prevalence (Wooten, 2004; Bourque et al., 2009; Hirsch et al., 2016; Meoni et al., 2020; Turcano and Savica, 2020), later onset (Haaxma et al., 2007; Meoni et al., 2020), and better motor scores in the Unified PD Rating Scale (UPDRS; Meoni et al., 2020). The influence of biological sex and gonadal hormones on dopamine neurodegeneration (Xing et al., 2017), neuroinflammation (Villa et al., 2016) and oxidative stress (Chainy and Sahoo, 2020) could explain the apparent less susceptibility and milder progression of motor symptoms in women (Cerri et al., 2019). However, how hormones modify PD features in women during menopause and how this compares to men needs further elucidation.

To understand if gonadal hormones play a role in the sexually dimorphic clinical presentation and response to treatment in PD patients (Georgiev et al., 2017), many studies have shown a link with total lifetime exposure to circulating sex hormones (Gatto et al., 2014), reproductive life events that alter ovarian hormone levels, like menopause and pregnancy (Martignoni et al., 2003) and hormone targeted therapies such as use of oral contraceptives (Cartwright et al., 2016) and hormone replacement therapy (HRT; Wang et al., 2015). Altogether, the current clinical literature points toward a trend on a neuroprotective effect of estrogen in PD (Tsang and Ho, 2001); however, inconsistencies between findings have been reported. In this review, we aimed to expand the current understanding of the hormone-PD link observed in patients by discussing the current and most updated clinical evidence. Specifically, we focus on three associations: age at menopause-age at PD onset, type of menopause-PD risk, and HRT use-PD risk. Further, we discuss the possible sources of discrepancies among studies, which meta-analysis studies may not be able to highlight.

### Criteria for literature selection

We exhaustively covered the clinical literature, published until July 2021, focusing on the association between menopause and HRT with PD risk using the following key words: Menopause, Early Menopause, Premature Menopause, Perimenopause, Hormone replacement therapy, estrogen, progesterone, AND/OR Parkinson’s disease, Neurodegenerative diseases, in PubMed and MEDLINE databases. The articles included in the main analysis of this review are shown in Table 1. Articles that did not assess the risk of PD or had incomplete data were excluded from the main discussion. Only papers with reported adjusted odds or relative risk ratios (OR/RR) were included in the figures.

**Table 1:** Observational studies on menopause and HRT and the association with risk of PD included in the main text.

Study	Type of study	PD sample size	Reproductive factors assessment type	Type of menopause	Multivariate/ Matched adjustment	HRT	HRT duration
Ascherio et al., 2003	Cohort	154	Medical record Self-reported	Natural Hysterectomy ≤1 oophorectomy Bilateral oophorectomy	(1), (3), (4), (7), (8), (9), (10)	HRT	<5 years ≥5 years
Baldereschi et al., 2003	Cohort	113	In-person interview	N/A	(1), (3), (5), (13)	ERT	N/A
Benedetti et al., 2001	Case–control	72	Medical record	Natural Surgical Hysterectomy only Bilateral oophorectomy	(5), (7)	ERT	< 6 months ≥ 6 months
Canonico et al., 2021	Case–control	130	Medical record In-person interview	Natural Hysterectomy Bilateral oophorectomy	(1), (3), (4), (5), (7), (9), (13), history of head trauma.	HRT	N/A
Cereda et al., 2013	Cross-sectional	497	Self-reported In-person interview	Natural Surgical	(1), (3), (5), (6), (12), (13) diabetes, hypertension, NSAID use, sedentary lifestyle, birth cohort and regular menses and the clinical features of PD.	HRT	>6 months
Currie et al., 2004	Case–control	68	Self-reported In-person interview	Natural	(1)	ERT	N/A
Frentzel et al., 2017	Cross-sectional	54	Medical record Self-reported	Natural	N/A	HRT	N/A
Gatto et al., 2014	Case–control	228	Self-reported	Natural Hysterectomy ≤1 oophorectomy Oophorectomy	(1), (2), (3)	HRT ERT	N/A
Greene et al., 2014	Case–control	743	Medical record Telephone interview	Natural Bilateral oophorectomy	(1), (3), (4), (5), (14), (15), (16)	HRT	≥ 5 years
Kim et al., 2021	Cohort	2,313	Medical records	N/A	(1), (2), diabetes, hypertension, cardiovascular disease, chronic kidney disease.	HRT	≤1 year 1–3 years 4–6 years >6 years
Kusters et al., 2021	Case–control	805	Medical record In-person interview	Natural Bilateral oophorectomy	(1), (3), (4), (5), (6), (7), (8)	HRT	N/A
Liu et al., 2014	Cohort	410	Self-reported	Natural Hysterectomy ≤1 oophorectomy	(1), (2), (3), (4), (7), (8), (10)	HRT ERT ERT + PRT	1–9 years ≥10 years
Lundin et al., 2014	Case–control	137	Medical records	N/A	(2), (3), (4), (5), (15),	HRT ERT ERT + PRT	≥ 2 years
Martignoni et al., 2003	Case–control	150	In-person interview	Natural Surgical	N/A	HRT	N/A
Nicoletti et al., 2011	Case–control	200	Self-reported	Natural Hysterectomy	(1), (3), (4), (15)	HRT	≥ 6 months
Nitkowska et al., 2014	Case–control	76	Medical record	Natural Surgical	N/A	N/A	N/A
Popat et al., 2005	Case–control	178	Medical record In-person interview	Natural Hysterectomy Hysterectomy ≥1 oophorectomy	(1), (3), (7), (8), (12)	HRT	1–10 years > 10 years
Ragonese et al., 2004	Case–control	131	Self-reported	Natural Surgical	(1), (3), (4), (5), (9)	ERT	≥ 6 months
Ragonese et al., 2006	Cross-sectional	145	Medical record	Natural Surgical	(3), (5), (8)	N/A	N/A
Rocca et al., 2008	Cohort	79	Medical record Self-reported In-person interview	Hysterectomy ≤1 oophorectomy Bilateral oophorectomy	(1), (5)	ERT	N/A
Rugbjerg et al., 2013	Cohort	77	Self-reported	Natural Hysterectomy Oophorectomy	(1)	HRT	N/A
Simon et al., 2009	Cohort	244	Self-reported	Natural Hysterectomy ≤1 oophorectomy Hysterectomy >1 oophorectomy	(1), (3), (4)	ERT PRT ERT + PRT	< 5 years ≥ 5 years
Yadav et al., 2012	Case–control	81	In-person interview	Natural Surgical	(1)	ERT	N/A
Yoo et al., 2020	Cohort	varies	Medical record, Self-reported	Natural Surgical (excluding hysterectomy +/− oophorectomy)	(3), (7), (9), (11), exercise, hypertension, diabetes, cancer, dyslipidemia.	HRT	< 2 years, 2–5 years, ≥ 5 years

Matching or multivariate adjustments were (1) age, (2) race, (3) smoking, (4) coffee/caffeine, (5) education, (6) positive familial history, (7) age at menopause, (8) type of menopause, (9) alcohol, (10) oral contraceptives, (11) parity, (12) respondent type, (13) pesticide-use, (14) degree of urbanization, (15) family PD history, (16) age at first symptom. N/A, not available; HRT, hormone replacement therapy; ERT, estrogen replacement therapy; PRT, progesterone replacement therapy.

### Age at menopause-age at PD onset and risk of PD

The lower prevalence of PD in women compared to men (Gillies et al., 2014; Picillo et al., 2017; Marras et al., 2018; Meoni et al., 2020) suggests that reproductive life milestones, such as pregnancy, age at menopause, and duration of fertile life may protect women from greater PD deterioration (Martignoni et al., 2002; Miller and Cronin-Golomb, 2010; Gillies et al., 2014), since these determine the cumulative lifetime exposure to endogenous estrogens (Gatto et al., 2014; Yoo et al., 2020). Average age at onset in PD is ~60–65 years old (Poewe et al., 2017). From a clinical perspective, the closest reproductive life event to PD onset is the menopause transition (perimenopause), a period of approximately 5 years affecting women of ~45–55 years old. Perimenopause is typified by erratic fluctuations in hormone levels (Brinton et al., 2015) during a life stage that coincides with the PD prodromal phase (~10–20 years before clinical symptoms emerge). This terminates in a post-menopause stage characterized by low levels of ovarian hormones, estrogen, and progesterone (Harlow et al., 2012). The age at menopause is defined as the age at which a woman has had amenorrhea for 12 months ending the menopausal transition (Harlow et al., 2012). A higher occurrence of PD in post-menopausal versus pre-menopausal women has been reported (Ragonese et al., 2004; Ragonese et al., 2006; Labandeira-Garcia et al., 2016; Lv et al., 2017; Picillo et al., 2017; Jurado-Coronel et al., 2018). Hence, a clear understanding of recommendations, follow-ups, and therapies is needed for physicians treating patients during the perimenopause transition. Nevertheless, current PD treatments follow a one-size-fits-all approach and do not take sex and menopausal stage into account. In the next paragraphs we will highlight the main literature in support of a positive association between menopause and PD risk and age at onset.

**Figure 1:**
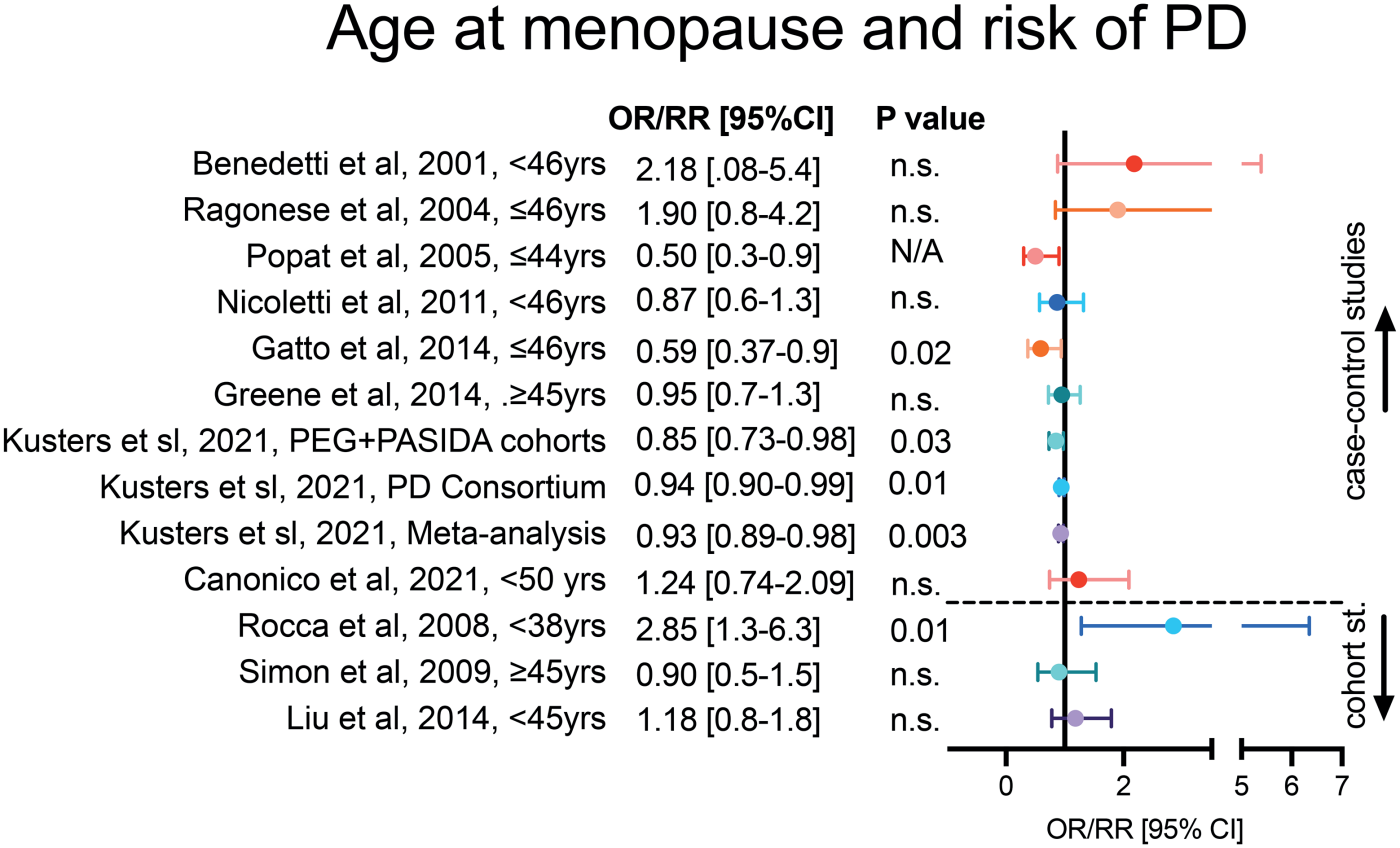
Age at menopause and risk of PD onset. Case–control studies in the upper section and cohort studies in the lower section of the forest plot are represented. For each study, figure reports adjusted OR/RR and 95% CI with level of statistical significance (*p* value). OR = odds ratio, RR = relative risk, 95% CI = 95% confidence of interval.

We found 16 observational studies that have assessed the association between menopause and PD in the last two decades. 10 case–control, 3 longitudinal cohort and 3 cross-sectional studies (Benedetti et al., 2001; Ragonese et al., 2004; Popat et al., 2005; Ragonese et al., 2006; Nicoletti et al., 2007; Rocca et al., 2008; Simon et al., 2009; Yadav et al., 2012; Cereda et al., 2013; Gatto et al., 2014; Greene et al., 2014; Liu et al., 2014; Nitkowska et al., 2014; Frentzel et al., 2017). 7 case–control studies analyzed the risk of PD in women with early age at menopause (Figure 1). Among these, Benedetti et al. (2001) and Ragonese et al. (2004) have reported higher odds of PD onset in women which reached menopause before 46 years-old. However, the adjusted multiple logistic regression models in these studies did not reach statistical significance. The recent work by Canonico et al. showed a significant association between age at menopause <50 years-old and risk of PD (Canonico et al., 2021). Another report by Nitkowska et al. (2014) showed that while early menopause occurred in only 16% of the control subjects, this number increased to 24% in the PD cohort. Other cohort studies have explored the relationship as well between age at menopause and PD risk. Liu et al. (2014) reported increased odds of PD onset in women with early menopause (<45 years-old). Similarly, Simon et al. (2009) showed a trend toward a decreased risk of PD in women with menopause after 45 years-old, nevertheless these studies did not reach significance. Interestingly, in the cohort study from Rocca et al. (2008), a prominently higher and significant risk of PD was reported in women with premature menopause (<38 years-old) compared to women with early menopause (38–45 years old; Figure 1).

In accordance with case–control reports, cohort studies do not always reach statistical significance. Potential points of discrepancy between studies may be the unstandardized research criteria used to classify patients, regression adjustment criteria, and formal representation of the analyses. As an example, the cohort study by Rocca et al. (2008) reported that menopause occurring before 38 years-old is an independent risk factor for PD; however the study analyzed the risk only in women with history of oophorectomy, in contrast to the other 2 mentioned cohort studies by Liu et al. (2014) and Simon et al. (2009), which included patients with natural and surgical menopause. Although these 2 studies were similar (Simon et al., 2009; Liu et al., 2014), the regression analyses were adjusted for different covariates leading to a difficult comparison. Additional support for a significant association between late age at menopause and decrease risk of PD comes from a recent work by Yoo et al., although the data was expressed as a Hazard Ratio rather than OR/RR and patients with history of hysterectomy were excluded from the study (Yoo et al., 2020).

Interestingly, to reduce certain biases common in observational studies, a recent work by Kusters et al. proposed the use of Mendelian randomization (MR) analyses to address the association of menopause age and PD risk (Kusters et al., 2021). The authors applied a MR to identify genetic variants linked to menopause and PD and used the 8 identified single nucleotide polymorphisms as an instrumental variable to demonstrate a significant inversed association between menopause age and risk of PD (Kusters et al., 2021). This suggests that non modifiable factors, such as genetic variants, in concomitance with the menopause might influence the risk of PD.

One case–control (Yadav et al., 2012), and 3 cross-sectional (Ragonese et al., 2006; Cereda et al., 2013; Frentzel et al., 2017) studies have explored the linear association between age at menopause and age at PD onset. The cross-sectional studies conducted by Yadav et al. (2012) and Frentzel et al. (2017) reported a significant positive correlation. Yadav et al. showed a positive correlation between age at menopause and age at PD onset (R = 0.55, *p* = 0.001), analyzing age-matched PD and healthy females in their case–control study (Yadav et al., 2012). Whereas, Frentzel et al. (2017) analyzed age-matched PD females and males, reporting also a positive correlation between age at menopause and age at PD onset (beta = 0.370, *p* < 0.01, adjusted R^2^ = 0.121). Likewise, Ragonese et al. (2006) (beta = 0.25, SE = 0.15, *p* = 0.003) and Cereda et al. (2013) (Coeff. = 13.03, SE =5.62, *p* = 0.021). Despite the heterogeneity in sample size and inclusion criteria of these 4 studies, the authors analyzed the association between age at PD onset and age at menopause using numerical variables instead of binary categorizations, which led to more consistent and statistically significant results.

### Type of menopause and risk of PD

To better understand how changes in levels of endogenous estrogen in menopausal women are associated with PD, previous works have looked at risk of PD in women with a history of surgical menopause. These studies suggested that abrupt decline of estrogen in women undergoing hysterectomy and uni/bilateral oophorectomy, commonly termed surgical menopause, may lead to a higher risk of PD, compared to women that experience a natural gradual change in estrogen levels during the menopause transition. However, the current evidence on this matter is in part inconsistent. In fact, it has been suggested that perimenopause acts as a neurological transition period, rendering the brain particularly susceptible to neurodegeneration (Brinton et al., 2015). Thus, the debate on whether the risk of PD neurodegeneration is triggered by the abrupt and complete loss of ovarian hormones, in the surgical menopause, or by erratic hormonal fluctuations during a critical window of time, such as the perimenopause, is still very active.

**Figure 2:**
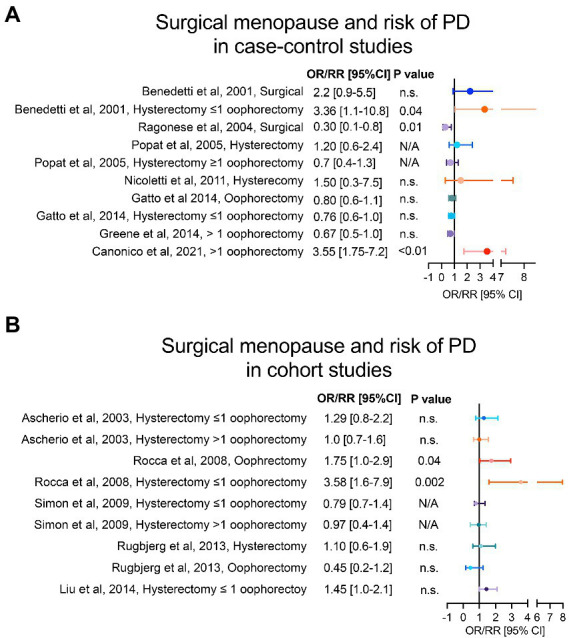
Type of menopause and risk of PD. Panel **(A)** shows case–control studies and panel **(B)** cohort studies that have analysed the risk of PD in women with surgical menopause compared to women with natural menopause. For each study, we reported the adjusted OR/RR and 95% CI with level of statistical significance (p value). OR = odds ratio, RR = relative risk, 95% CI = 95% confidence of interval.

Among the clinical studies that have investigated the role of menopause in PD, case control studies by Benedetti et al. (2001) and Ragonese et al. (2004) have found opposite association between risk of PD onset and surgical menopause, although the type of surgical menopause was not specified. Interestingly, the risk of PD onset seems to be linked to the type of surgical menopause performed. In Canonico et al. a significant association between bilateral oophorectomy, but not hysterectomy, and risk of PD was found (Canonico et al., 2021). The prevalence of bilateral oophorectomy in controls was 9% in comparison to nearly 25% in PD cases. Furthermore, hysterectomy performed before 45 years-old increases the risk of PD onset, as reported by Nicoletti et al. (2011) and Popat et al. (2005) and in the cohort study by Rugbjerg et al. (2013). However, the findings were not significant in adjusted models. Divergent results have been reported regarding hysterectomy combined with unilateral oophorectomy. While some reports (Ascherio et al., 2003; Simon et al., 2009; Gatto et al., 2014; Liu et al., 2014) indicate no association with the risk of PD onset, studies by Benedetti et al. (2001) and Rocca et al. (2008), reported a significant risk of PD onset (up to three-fold higher). Instead, hysterectomy combined with bilateral oophorectomy has shown a protective effect in most case control and cohort studies (Ascherio et al., 2003; Popat et al., 2005; Simon et al., 2009; Rugbjerg et al., 2013; Gatto et al., 2014; Greene et al., 2014), with the exception of Rocca et al. (2008). Altogether, these studies point towards an increased risk of PD onset when hysterectomy is combined with unilateral oophorectomy and, at younger age (Figures 2A,B).

Despite the studies mentioned above suggest an increased risk of PD in women who underwent surgical menopause, compared to those who experienced a physiological menopause, the evidence about type of menopause (i.e., natural vs. surgical) and its relation to the risk of PD onset remains conflicting. The source of inconsistent findings between these studies might be related to important confounding factors. Particularly, the underlying condition that prompts the surgical indication of hysterectomy and/or oophorectomy, and the medical management of the different type of menopause. The most common indications for hysterectomy are leiomyoma and abnormal uterine bleeding, known to be deeply related to progesterone and estrogen abnormalities (Vilos et al., 2015; Jewson et al., 2020). Hence, even though both ovaries are preserved in this surgical procedure, this is preceded by hormonal dysfunctions (Torrealday et al., 2017). When bilateral oophorectomy is performed, with or without uterus resection, women commonly receive preventive exogenous gonadal hormones (Domchek and Rebbeck, 2007). This may be a confounding factor when evaluating the association between bilateral oophorectomy and risk of PD onset and may explain the protective trend observed in some studies. Regarding the increased risk found for hysterectomy combined with unilateral oophorectomy, previous evidence has shown ovarian failure in the contralateral ovary following unilateral oophorectomy (Farquhar et al., 2005), paralleled by a loss of blood supply to the remaining ovary due to the uterus resection (Ahn et al., 2002). Furthermore, although higher risk of early ovarian failure has been reported in patients with history of unilateral oophorectomy (Rosendahl et al., 2017), most of these women do not receive HRT (Read et al., 2010). Finally, it’s worth mentioning that a possible age-dependent effect, found in linear trend analyses of age at surgical menopause and risk of PD onset (Rocca et al., 2008), adds more variability to the mentioned findings. This suggests that age stratification should be analyzed in more depth in future studies.

### Hormone replacement therapy and risk of PD

The molecular weight of endogenous gonadal hormones allows easy diffusion across the blood–brain barrier (Diotel et al., 2018). Likewise for exogenous steroids (Cipolla et al., 2009), although their preventive or detrimental potential on the neurons remains unclear (Simpkins et al., 2005). Exogenous steroids, also known as HRT, are commonly prescribed to women to relieve menopausal symptoms (Valdes and Bajaj, 2020). Conventional HRT includes both estrogen and progesterone hormones with various formulations and exerting different specificity of effects on the gonadal-brain axis (Schipper, 2016; Del Rio et al., 2018).

**Figure 3:**
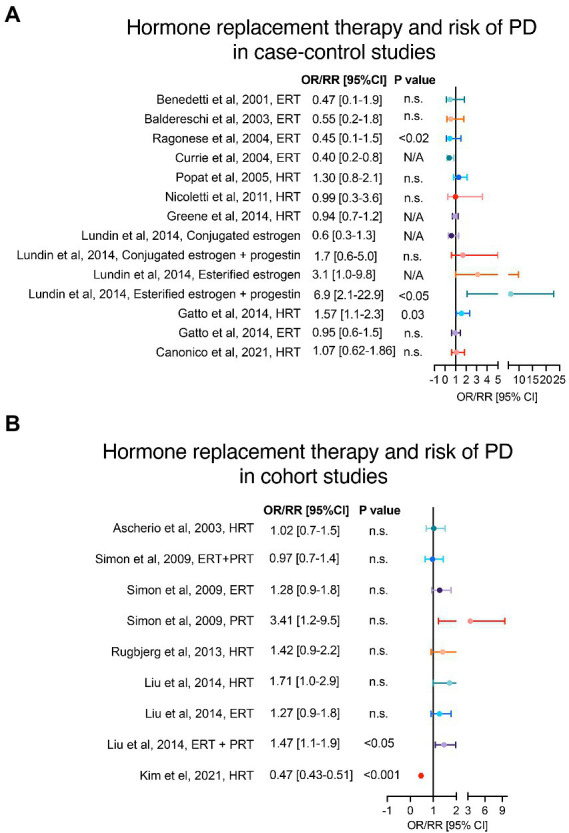
Hormone replacement therapy and risk of PD. Panels **(A,B)** show case–control and cohort studies, respectively, that have analysed the risk of PD in women with history of HRT. Adjusted OR/RR and 95% CI with level of statistical significance (*p* value). OR = odds ratio, RR = relative risk, 95% CI = 95% confidence of interval, HRT = hormone replacement therapy, ERT = estrogen replacement therapy, PRT = progesterone replacement therapy.

Several studies have explored the role of HRT on the risk of PD onset. A trend toward an increased risk of PD onset in women that received HRT, without distinction of formulation type, was observed in case–control and cohort studies (Ascherio et al., 2003; Martignoni et al., 2003; Popat et al., 2005; Nicoletti et al., 2007; Simon et al., 2009; Rugbjerg et al., 2013; Greene et al., 2014; Liu et al., 2014), as shown in Figures 3A,B. However, only in the study from Gatto et al. (2014) the results reached statistical significance in adjusted models. A more detailed assessment of the HRT formulations evidences a modest increased risk of PD in users of estrogen replacement therapy (ERT) in 2 cohort studies (Simon et al., 2009; Liu et al., 2014) in contrast to 6 case–control studies (Benedetti et al., 2001; Baldereschi et al., 2003; Currie et al., 2004; Ragonese et al., 2004; Gatto et al., 2014; Lundin et al., 2014) that showed a trend for a protective or no effect of ERT. The discrepancies between cohort and case–control studies may be explained by lack of analyses regarding the age at therapy initiation, type or stage of menopause, as well as the ERT subtype. Importantly, the combination of ERT with progesterone or progesterone-like replacement therapy (PRT) in Liu et al. (2014) and Lundin et al. (2014) showed a significant increased risk of PD onset. Moreover, Simon et al. (2009) reported higher odds of PD risk in a small sample of women receiving PRT alone compared to women receiving ERT alone or combined therapy.

In support of this, a recent retrospective analysis by Kim et al. showed that sources of discrepancy in the effect of HRT on different neurodegenerative disorders, including PD, may be related to route and duration of HRT administration (Kim et al., 2021), whereas a significantly reduced relative risk (RR) of PD was reported in women taking oral, but not transdermal, therapy. Additionally, this work supports the importance of including large sample sizes in this type of studies (Kim et al., 2021).

Regarding the ERT findings, it is important to highlight that there are two common formulations: the esterified estrogens and the conjugated estrogens. The esterified estrogens are predominantly estrone, whereas conjugated estrogens are a mixture of more biologically active estrogens (like 17b-estradiol) with greater affinity for estrogen receptors than estrone (Lundin et al., 2014); nevertheless, these two formulations share the same FDA indications (Harper-Harrison and Shanahan, 2020). Lundin et al. (2014) showed that the trend toward an increased or decreased risk of PD onset is inverse depending on whether esterified or conjugated formulations are administered. Another interesting observation in the Benedetti et al. (2001) study was the opposite ERT contribution to PD in women depending on the menopausal type (i.e., natural vs. surgical). This association was supported by a similar observation in the study by Popat et al. (2005) in which women with natural menopause that received HRT had lower odds of developing PD, while women with oophorectomy plus hysterectomy on HRT had higher risk of PD onset. Additionally, studies on dementia and Alzheimer’s disease, like the one by Whitmer et al. (2011), support the “window of opportunity hypothesis” that the use of HRT in midlife (before or during early menopause) only may be neuroprotective, whereas HRT initiation in late life could have deleterious effects and worsen the neurodegenerative processes (Marras and Saunders-Pullman, 2014; Kim and Brinton, 2021). Thus, we suggest that a stratification analysis of age at HRT initiation may clarify discrepancies seen in previous clinical observational studies and, moreover, could shed lights on possible age/time-dependent mechanisms of hormones in the central nervous system.

Although the publication of the Women’s Health Initiative data in 2002 supported that HRT increases the risk of stroke and breast cancer (Rossouw et al., 2002), a recent national survey study reported that 37% of women are current or former HRT users (Gass et al., 2015). Therefore, a rigorous assessment of the HRT doses and different formulations in regard to the type of menopause, age of menopause, duration of HRT, medical indication of HRT, and other factors that can interact with HRT, such as caffeine consumption (Kim et al., 2017), is needed to improve recommendations for women in menopause. Even more, evidence from some of the mentioned observational studies regarding HRT suggests that estrogens may not be the only gonadal hormones capable of affecting the course of PD. Hence, the role of progesterone in influencing the central nervous system directly, through its neuronal receptors, or indirectly, through its action on the peripheral systems, requires further elucidation (Bourque et al., 2019; Cardia et al., 2019; Jarras et al., 2020; Kim et al., 2021).

In Lundin et al. the type of progestin used was the synthetic progesterone formulation medroxy-progesterone acetate (MPA), whereas this was not specified in Liu et al. and Simon et al (Simon et al., 2009; Liu et al., 2014; Lundin et al., 2014). Pre-clinical cell and animal models have shown progesterone to be neuroprotective, but not MPA (Singh and Su, 2013). Preclinical data suggest that progesterone may be neuroprotective in PD by increasing dopaminergic neurotransmission, exerting anti-inflammatory activity, and modulating several other neurotransmitter systems (including glutamatergic, GABAergic, norepinephrine, serotonin, and acetylcholine; Callier et al., 2001; Kritzer et al., 2003; Casas et al., 2013; Barth et al., 2015; Litim et al., 2017). Differently from endogenous progesterone, MPA is detrimental to neurons as it can induce glutamate toxicity and counteract the neuroprotective and neurotrophic effects of 17beta-estradiol (E2; Nilsen et al., 2006; Singh and Su, 2013). This is of particular importance as MPA is often the progestin used in HRT (Kim et al., 2021) and could therefore explain the trend towards increased risk of PD observed in the 3 abovementioned studies (Simon et al., 2009; Liu et al., 2014; Lundin et al., 2014).

### Future perspectives and conclusions

Throughout this review, we have highlighted that within the same type of studies the conflicting evidence underlines the different methods of data collection, patient’s classification, and regression models. Similarly, in different types of observational studies the discrepancies may relate to bias in the population inclusion criteria and sample size. Our work emphasizes the importance of considering a uniform standard criterion to adjust regression models with a consistent statistical and clinical judgment. We believe that despite some inconsistent results, the current findings support a role for menopause on the risk of PD onset. This is an exciting research field for scientists working in basic, pre-clinical and clinical sciences aiming to elucidate the underlying mechanisms in PD and promoting better strategies to manage menopausal patients accordingly to their risk profile.

The effect of gonadal steroids on the brain dopamine system has been the subject of numerous pre-clinical publications in the past several decades. Pre-clinical studies have thus far led the way to elucidate the effect of gonadal steroids more consistently on dopamine containing neurons in wild type and parkinsonian animals, especially in toxin-based rodent models of PD (Dluzen et al., 1996; Miller et al., 1998; Grandbois et al., 2000; Quinlan et al., 2013; Gillies et al., 2014; Smith and Dahodwala, 2014; Almey et al., 2015; Rodriguez-Perez et al., 2015; Almey et al., 2016; Labandeira-Garcia et al., 2016; Jurado-Coronel et al., 2018). Several works support the hypothesis that menopause may constitute a triggering risk factor, which interaction with other risk factors and other possible pathological processes may modify the onset of PD. For instance, pre-clinical studies using rotenone and MPTP toxin-induced animal models of PD showed that ovariectomy abolishes the neuroprotective advantage observed in the substantia nigra and striatum of females as compared to males with PD. Conversely, treatment with estrogen reduces dopaminergic neurodegeneration in the substantia nigra and restores dopaminergic transmission (Disshon and Dluzen, 2000; Mitra et al., 2015; Shen et al., 2017; Makav and Eroglu, 2021). Similarly, clinical studies are starting to elucidate the combined effect of menopause with other PD risk factors. Among postmenopausal women, sleep disturbances were associated with approximately 10–30% increased PD risk after ∼16 years follow-up; although prospective cohort studies that include both men and women of diverse backgrounds are required to confirm these findings (Beydoun et al., 2022).

Other nuances important to mention include the notion that, as suggested by the considerable number of studies reviewed in this article, the physiological changes and pathological mechanisms involved in PD neurodegeneration may adapt distinctively at different stages of the menopause process. Moreover, recent studies have approached the concept that perimenopause does not equal the simple loss of estrogen, but that it represents a period of gonadal endocrine imbalance and neurological transition during which the nigro-striatal circuit is more susceptible to PD neurodegeneration (Brinton et al., 2015).

## Author contributions

SU, SM, RM, and TM: conceptualization, methodology, resources, investigation, writing—draft preparation, review, and editing. RM and TM: funding acquisition. RM: project administration. All authors contributed to the article and approved the submitted version.

## Funding

This research was funded in whole or in part by the Aligning Science Across Parkinson’s (ASAP-020608; RM) through the Michael J. Fox Foundation for Parkinson’s Research (MJFF), the American Parkinson’s Disease Association (APDA 190167-01; RM), and the National Institute of Health grants R21AG064455 (RM), HL098351 (TM), and DA08259 (TM).

## Conflict of interest

The authors declare that the research was conducted in the absence of any commercial or financial relationships that could be construed as a potential conflict of interest.

## Publisher’s note

All claims expressed in this article are solely those of the authors and do not necessarily represent those of their affiliated organizations, or those of the publisher, the editors and the reviewers. Any product that may be evaluated in this article, or claim that may be made by its manufacturer, is not guaranteed or endorsed by the publisher.
